# Effects of *Tricholoma* Matsutake-Derived Insoluble Fiber on the Pasting Properties, Structural Characteristics, and In Vitro Digestibility of Rice Flour

**DOI:** 10.3390/foods14122143

**Published:** 2025-06-19

**Authors:** Qin Qiu, Jing Chen, Dafeng Sun, Yongshuai Ma, Yujie Zhong, Junjie Yi, Ming Du, Man Zhou, Tao Wang

**Affiliations:** 1 Faculty of Food Science and Engineering, Kunming University of Science and Technology, Kunming 650500, Chinamays186@163.com (Y.M.);; 2 Key Laboratory of Plateau Characteristic Prepared Food in Yunnan Province, Kunming 650500, China; 3 International Green Food Processing Research and Development Center of Kunming City, Kunming 650500, China; 4 Kunming Edible Fungi Institute of All China Federation of Supply and Marketing Cooperatives, Kunming 650221, China; sundf1977@outlook.com; 5 School of Food Science and Technology, Dalian Polytechnic University, Dalian 116034, China; 6 School of Food and Biological Engineering, Jiangsu University, Zhenjiang 212013, China

**Keywords:** *Tricholoma matsutake*, insoluble dietary fiber, starch gelatinization, in vitro digestion

## Abstract

This study explores the effects of *Tricholoma matsutake*-derived insoluble dietary fiber (TMIDF) on the pasting behavior, structural properties, and in vitro digestibility of rice flour. The incorporation of 5% TMIDF significantly increased the peak viscosity (from 2573.21 to 2814.52 mPa·s) by competitively adsorbing water and forming a dense transient network, while simultaneously reducing the final viscosity (from 1998.27 to 1886.18 mPa·s) by inhibiting amylose recrystallization. Multi-scale structural analyses revealed that TMIDF enhanced V-type crystallinity and limited enzyme access via a porous fibrous matrix. Fourier-transform infrared spectroscopy and low-field nuclear magnetic resonance analyses confirmed that hydrogen bonding and water redistribution were key interaction mechanisms. TMIDF significantly lowered in vitro starch digestibility and increased resistant starch content by 16% (from 14.36% to 30.94%) through synergistic effects, including physical encapsulation of starch granules, formation of enzyme-resistant amylose-lipid complexes, and α-amylase inhibition (31.08%). These results demonstrate that TMIDF possesses a unique multi-tiered modulation mechanism, involving structural optimization, enzyme suppression, and diffusion control, which collectively surpasses the functional performance of conventional plant-derived insoluble dietary fibers. This research establishes a theoretical basis for applying fungal insoluble dietary fibers to develop low glycemic index functional foods, highlighting their dual role in improving processing performance and nutritional quality.

## 1. Introduction

Rice, as an essential staple crop on a global scale, accounts for 70–80% of the caloric intake in many populations. Its predominant component, starch, acts as both a vital metabolic substrate and a key hydrocolloid for texture engineering in food processing systems [1]. Despite its widespread use, native starch exhibits critical processing constraints, including low shear tolerance, compromised thermomechanical integrity, and predisposition to post-processing structural retrogradation. These factors restrict its application in various food manufacturing processes [2]. Among the functional properties of starch, digestibility has garnered considerable attention due to its direct impact on glucose release kinetics, postprandial glycemic response, and the associated risk of type 2 diabetes mellitus (T_2_DM) [3]. Consequently, developing low glycemic index (GI) foods has become a priority in contemporary nutrition research [4,5]. This focus has driven systematic investigations into a comprehensive array of modification strategies, spanning physical processing techniques, chemical treatments, and biological modification approaches. Notably, the physical blending of starch with hydrocolloids or non-starch polysaccharides (e.g., dietary fibers) offers a viable route to enhance both the starch processing performance and its nutritional attributes, owing to its eco-friendliness and multifunctionality [6,7].

Dietary fiber (DF) is well-recognized for its broad spectrum of health-promoting effects, particularly its positive influence on metabolic health [8]. Insoluble dietary fiber (IDF), specifically, plays a crucial role in enhancing intestinal motility, adsorbing cholesterol, and delaying glucose absorption [9]. In recent years, there has been a growing interest in incorporating DF into starch-based systems to improve both the functional properties and nutritional quality of starch. For instance, wheat bran IDF has been demonstrated to inhibit α-amylase activity, thereby reducing starch digestibility [10], while soluble dietary fiber (SDF) from rice bran has been found to suppress long-term starch retrogradation and elevate the gelatinization temperature [11]. Furthermore, adjusting the SDF/IDF ratio has been found to enhance noodle quality and attenuate starch hydrolysis [12]. Despite these advancements, most studies have primarily focused on elucidating the interaction mechanisms between plant-derived IDFs (e.g., oat, inulin) and starch [13,14]. The regulatory roles of IDFs derived from medicinal-edible fungi, such as *Tricholoma matsutake*, remain largely unexplored.

*T. matsutake*, a renowned medicinal and edible fungus, is rich in bioactive compounds, such as polysaccharides, proteins, and polyphenols, which exhibit antioxidant, anti-inflammatory, and digestion-regulating properties [15]. In addition to these bioactive components, *T. matsutake* contains a significant amount of insoluble dietary fiber in its fruiting bodies. The *T. matsutake*-derived insoluble dietary fiber (TMIDF) may interact with starch through various physicochemical mechanisms. Previous studies on cereal-derived IDFs, such as wheat bran, have shown that these fibers can influence starch gelatinization behavior by enhancing molecular chain entanglement and overlap by non-covalent interactions [16]. Furthermore, IDFs can physically encapsulate starch granules, helping preserve short-range ordered structures such as the double helices of starch [17]. Notably, enzymatically extracted IDFs retain their structural integrity and bioactive components, making them ideal for use in food applications [18]. Based on these findings, we hypothesize that TMIDF may significantly influence the gelatinization properties and physicochemical characteristics of rice flour (RF) through mechanisms such as competitive water adsorption and non-covalent interactions. Furthermore, it could potentially reduce post-gelatinization digestibility by acting as a physical barrier or inhibiting enzyme activity.

This study aims to investigate the structural and functional effects of TMIDF incorporation (5% *w*/*w*) on RF through comprehensive analyses of pasting properties, dynamic rheological behavior, thermal transitions, and microstructural characteristics. Furthermore, the inhibitory effects of TMIDF on α-amylase activity and its modulation of starch digestibility will be elucidated. A range of multiscale characterization techniques, including low-field nuclear magnetic resonance (LF-NMR), Fourier-transform infrared spectroscopy (FT-IR), X-ray diffraction (XRD), and thermogravimetric analysis (TGA), will be employed to elucidate the molecular interactions between TMIDF and starch. The findings from this study are expected to establish a theoretical foundation for the high-value utilization of TMIDF and advance the development of low GI functional foods.

## 2. Materials and Methods

### 2.1. Materials

Rice flour (RF), characterized by a moisture content of 4.1%, protein content of 6.2%, and free fat content of 0.7%, was obtained from Zou Youcai Food Co., Ltd. (Harbin, China). Fresh fruiting bodies of *T. matsutake* were obtained from a local agricultural market in Kunming, Yunnan Province, China. A glucose assay kit employing the GOPOD method was supplied by Nanjing Jiancheng Bioengineering Institute (Nanjing, China). Additionally, α-amylase (porcine pancreas origin, 9.4 U/mg) and acarbose (≥95% purity) were obtained from Yuanye Biotechnology Co., Ltd. (Shanghai, China). Amyloglucosidase (260 U/mL) and papain (200 U/mg) were purchased from Macklin Biochemical Co., Ltd. (Shanghai, China). Analytical-grade chemicals were used for all other reagents.

### 2.2. Extraction of TMIDF

The extraction of TMIDF was performed as per a previously reported method [10] with minor adjustments (primarily the reversal of the enzyme order). Briefly, the fresh *T. matsutake* samples were cleaned, freeze-dried, pulverized, and sieved through a 70-mesh sieve to obtain a fine powder. After homogenizing the powder in deionized water (1:10 *w*/*v*) and incubating for 15 min, centrifugation at 3600× *g* (10 min) was performed to discard the supernatant. The remaining precipitate was resuspended in deionized water, and its pH was adjusted to 6.0 using 1 mol/L citric acid. Subsequently, the suspension was treated with α-amylase (100 U/mL) at 60 °C for 90 min. Following this, papain (6000 U/mg) was added, and the mixture was incubated at 60 °C for an additional 60 min. Then, the pH was adjusted to 4.5, and amyloglucosidase (3000 U/mL) was added for hydrolysis at 55 °C for 30 min. Finally, all enzymatic reactions were terminated by boiling the solution for 15 min. After cooling to room temperature (~25 ± 2 °C), the resulting precipitate was collected via centrifugation (3600× *g*, 10 min), lyophilized, and milled to a fine powder. The powder was then passed through a 70-mesh sieve to obtain homogeneous TMIDF. The yield of the extraction process was 12.86%.

### 2.3. Preparation of Mixed RF-TMIDF

RF was blended with 5% (*w*/*w*) TMIDF. Deionized water was added to both the blended powder and pristine RF at a solid-to-liquid ratio of 1:10 (*w*/*v*). The mixture was homogenized by magnetic stirring (500 rpm, 30 min) to ensure even distribution of TMIDF. After homogenization, the mixture was freeze-dried to remove moisture and stored under dry conditions for further experimental use.

### 2.4. Rapid Viscosity Analysis (RVA)

The pasting properties of RF and the RF-TMIDF mixture were determined by Rapid Visco Analyzer (RVA, Rapid-20, Bosin Tech, Shanghai, China). Precisely 3 g of each sample was weighed into an RVA aluminum canister, mixed with 25 g of deionized water, and homogenized into a slurry. The RAV protocol initiated with a 1 min equilibration at 50 °C under high-shear mixing (960 rpm), followed by a temperature profile consisting of heating to 95 °C at 12 °C/min, holding for 5 min, cooling to 50 °C at the same rate, and a final 12.5 min equilibration. The entire operation was carried out under constant agitation at 160 rpm.

### 2.5. Dynamic Oscillation Rheology Measurement

Frequency sweep tests were conducted according to a previously established methodology [13]. Briefly, 3 mL of the RVA-gelatinized sample was rheologically assayed via an MCR 102 rheometer (Anton Paar GmbH, Graz, Austria) with 50-mm parallel plates (1-mm fixed gap). Dynamic oscillatory measurements were performed at 1% strain and 25 °C, covering an angular frequency range of 0.1–10 Hz. The storage modulus (G′) and loss modulus (G′′) were continuously monitored throughout the frequency sweep.

### 2.6. LF-NMR Measurement

The water distribution in the gels prepared in the RVA was measured using an LF-NMR analyzer (PQ001-20-020V, Niumag Analytical Instrument Co., Ltd., Shanghai, China). Approximately 3 g of each sample was loaded into a 10 mL transparent glass vial sealed with a screw cap. All measurements were conducted at ambient room temperature (25 ± 1 °C). The Carr-Purcell-Meiboom-Gill (CPMG) pulse sequence was employed to quantify spin-spin relaxation times (T_2_) and corresponding relative peak areas (P_2_) under the following acquisition parameters: 1024 echoes per scan with 8 cumulative scans.

### 2.7. DSC Analysis

The thermal characteristics of RF and RF-TMIDF were analyzed using a differential scanning calorimeter (DSC 7000, Seiko Instruments Inc., Chiba, Japan). Referring to the method of Wu [19] with slight modification. Samples (~2 mg) were placed in aluminum pans and weighed. Subsequently, 6 μL of deionized water was added, and the pan was hermetically sealed. The sealed samples were equilibrated at 25 °C for 8 h. Following equilibration, DSC analysis was performed using a heating program from 20 °C to 100 °C at 10 °C/min.

### 2.8. TGA

TGA was conducted using a thermogravimetric analyzer (TGA, Mettler Toledo, Greifensee, Switzerland) under a nitrogen atmosphere (20.0 mL/min flow rate). Samples were heated from 20 °C to 600 °C at 10 °C/min, with mass loss monitored during heating.

### 2.9. SEM Analysis

The microstructural features of freeze-dried samples prepared in Section 2.4 were characterized by a scanning electron microscope (SU-8010, Hitachi Ltd., Tokyo, Japan). The dried powder samples were adhered to conductive carbon tape and sputter-coated with a 10 nm gold-palladium layer to enhance surface conductivity. Observations were performed at an accelerating voltage of 3 kV under magnifications of ×500, ×1000, and ×2000.

### 2.10. FT-IR Spectroscopy Analysis

FTIR spectra of freeze-dried RVA gels were acquired on an IRTracer-100 spectrometer (Shimadzu, Kyoto, Japan), following the method described by a previous study [7]. Approximately 1 mg of each sample was intermixed with dried potassium bromide (KBr) at a ratio of 1:100, pelletized, and scanned over a wavenumber range of 4000–400 cm^−1^.

### 2.11. XRD Analysis

The crystallinity of freeze-dried RF-TMIDF and RF samples as loose powder was assessed by X-ray diffraction (DY5261, CEM Corp., Stallings, NC, USA). Data collection covered 5–40° 2θ with a 5°/min scanning speed.

### 2.12. α-Amylase Inhibition Rate

The α-amylase inhibitory activity of TMIDF was assessed following a modified protocol [20]. Briefly, 0.25 g of TMIDF powder was mixed with 1 mL of α-amylase solution (40 U/mL in 0.02 M PBS, pH 6.9) and incubated at 25 °C for 20 min. Following this, 1 mL of 1% soluble starch substrate was introduced, and the mixture was further incubated for 10 min. To terminate the reaction, 2 mL of 3,5-dinitrosalicylic acid (DNS) reagent was added. The terminated reaction mixture was then boiled for 5 min and cooled to room temperature (25 ± 2 °C). Absorbance at 540 nm was recorded using a BioTek Epoch2 microplate reader (Agilent, Santa Clara, CA, USA). Acarbose acted as the positive control at equivalent concentrations. The α-amylase inhibitory activity was calculated according to the following formula:

(1) $$\mathrm{Amylase}\;\mathrm{inhibition}\;\mathrm{activity}\left(\%\right)=\frac{\left(A_{0}-A_{1}\right)}{A_{0}}\times 100,$$

where *A*_0_ (control) and *A*_1_ (sample) are absorbance values.

### 2.13. In Vitro Starch Digestibility

The starch digestibility was evaluated with a slightly modified method of a previous study [21]. Following gelatinization, samples were equilibrated at 4 °C for 12 h, lyophilized, pulverized, and sieved (100-mesh). A 200 mg portion of the resulting powder was mixed with 15 mL phosphate buffer (0.5 M, pH 5.2) and 5 glass beads in a conical flask. Following 10 min of agitation at 37 °C, hydrolysis was initiated by adding 5 mL enzyme solution (containing 30 U/mL α-amylase and 20 U/mL amyloglucosidase). At designated time points (0, 5, 10, 20, 30, 45, 60, 90, 120, and 180 min), 0.1 mL aliquots were withdrawn, immediately transferred to centrifuge tubes preloaded with 0.9 mL ethanol to inactivate enzymes, and centrifuged (2500× *g*, 15 min). The supernatant’s glucose concentration was determined with a GOPOD assay kit. Subsequently, the contents of rapidly digestible starch (RDS), slowly digestible starch (SDS), and resistant starch (RS) were derived using standard equations [22].

(2) $$Starch\;digested\left(\%\right)=\frac{A\times 0.9}{B}\times 100,$$

(3) $$RDS\left(\%\right)=\frac{(G_{20}-G_{0})\times 0.9\times 100}{TS},$$

(4) $$SDS\left(\%\right)=\frac{\left(G_{120}-G_{20}\right)\times 0.9\times 100}{TS},$$

(5) $$RS\left(\%\right)=100-\left(RDS+SDS\right),$$

where variables A (mg) and B (mg) correspond to the initial starch mass and hydrolyzed glucose at reaction time t (min), respectively. The factor 0.9 reflects the starch-to-glucose molar conversion ratio. G0, G20, and G120 represent glucose content (g) released at 0, 20, and 120 min, respectively; TS indicates total starch content.

### 2.14. Statistical Analysis

Data are expressed as mean ± standard deviation (SD, *n* = 3). Statistical significances were analyzed using Student’s *t*-test, with significance set at *p* < 0.05. Statistical analysis was performed with Origin 9 software (OriginLab Corporation, Northampton, MA, USA).

## 3. Results and Discussion

### 3.1. Pasting Properties

**Figure 1:**
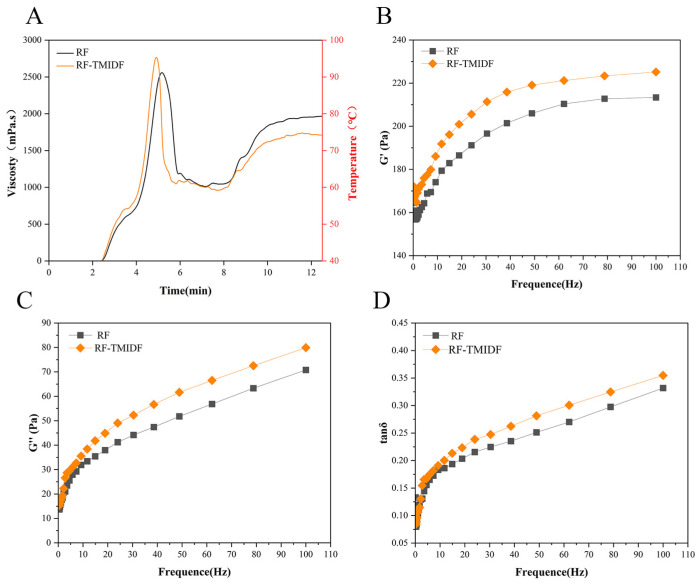
Comparative analysis of RF and RF-TMIDF gels. (**A**) viscosity-temperature profiles; (**B**) elastic response (G′); (**C**) viscous response (G′′); (**D**) viscoelastic ratio (tan δ).

As depicted in Figure 1A and Table 1, 5% TMIDF addition induced significant changes in RF pasting behavior. Compared to the native RF, the TMIDF-blended system (RF-TMIDF) displayed a notable increase in PV (from 2573.21 ± 31.29 mPa·s to 2814.52 ± 18.03 mPa·s, *p* < 0.05). indicating enhanced suspension stability during thermal processing. In contrast, the FV significantly decreased from 1998.27 ± 52.19 mPa·s to 1886.18 ± 31.74 mPa·s (*p* < 0.05). This seemingly contradictory trend can be explained by the dual action mechanisms of TMIDF: physical obstruction and molecular interactions. Specifically, TMIDF competes with starch granules for free water [23], thereby limiting the full hydration and swelling of starch. This constrained hydration environment forced starch granules to form a denser transient network during early heating, thereby enhancing shear resistance [24]. Simultaneously, the rigid TMIDF particles acted as structural fillers within the starch matrix, amplifying internal friction and further elevating the apparent viscosity of the system.

The SB decreased from 986.48 ± 21.70 mPa·s to 848.94 ± 47.27 mPa·s (*p* < 0.05). This decline potentially stems from the inhibitory effect of IDF particles on amylose diffusion and alignment during the cooling phase [25]. Additionally, hydrogen bonding between TMIDF hydroxyl groups and amylose chains may further reduce the availability of free amylose for retrogradation and recrystallization [26]. As a result, the combined effects of physical barriers and water competition led to 5.6% and 14% reductions in FV and SB, respectively (*p* < 0.05), which demonstrated the TMIDF’s efficacy in suppressing short-term starch retrogradation. This is a critical feature for enhancing the texture and shelf life of starch-based products.

Notably, the BD increased from 1558.09 ± 58.50 mPa·s to 1807.28 ± 9.22 mPa·s (*p* < 0.05), demonstrating a decline in the thermomechanical stability of the RF-TMIDF gel. This elevation in BD may be due to the heterogeneous distribution of TMIDF within the starch network. Under high-temperature and shear conditions, the rigid structure of TMIDF could disrupt the continuity of the gel, leading to a breakdown of the gel network and a subsequent collapse in viscosity [27]. Overall, these findings suggest that TMIDF not only modifies the hydration and pasting behavior of starch but also contributes to a more complex and potentially tunable rheological profile, with implications for designing functional food matrices with improved processing and storage stability.

### 3.2. Dynamic Rheological Properties

Frequency sweep tests characterizing the dynamic rheological properties revealed TMIDF’s influence on the viscoelastic behavior of the RF-TMIDF system (Figure 1). As shown in Figure 1B,C, the addition of TMIDF significantly increased both the storage modulus (G′) and loss modulus (G′′), reflecting an overall enhancement in the viscoelastic strength of the system enhanced viscoelastic strength of the composite system. These increases were accompanied by a rise in the loss tangent values (tanδ = G′′/G′, Figure 1D), suggesting a relative increase in the viscous component as compared to the elastic component.

Notably, all samples displayed G′ > G′′ across the 0.1–10 Hz frequency range, indicating that the RF-TMIDF system behaves as a weak gel with elastic-dominant characteristics [23]. However, the observed increase in both moduli (G′ and G′′) upon TMIDF addition indicates the formation of a more structured and interconnected gel network, potentially due to the physical entanglement and water-binding capacity of the insoluble fiber. The tanδ parameter, representing the ratio of viscous to elastic components, increased systematically with TMIDF addition. While all starch systems maintained tanδ < 1, the upward shift in tanδ values suggests a relative enhancement of viscous dominance, which aligns with the reduction in FV observed in RVA analysis (Table 1). This change suggests that although the addition of TMIDF strengthens the initial gel network, it also introduces heterogeneity in the water distribution and shear-sensitive microdomains within the starch matrix. These microdomains, which are sensitive to shear and temperature changes, may cause instability and compromise the overall thermodynamic stability of the starch network under dynamic deformation. These findings are consistent with the previous study on bamboo shoot IDF-modified RF systems [26], which similarly demonstrated that IDF addition modified water distribution and disrupted the uniformity of starch-based gels. Collectively, the results suggest that TMIDF not only reinforces the initial gel structure but also alters the internal rheological architecture, resulting in a more complex viscoelastic profile.

### 3.3. LF-NMR Analysis

**Figure 2:**
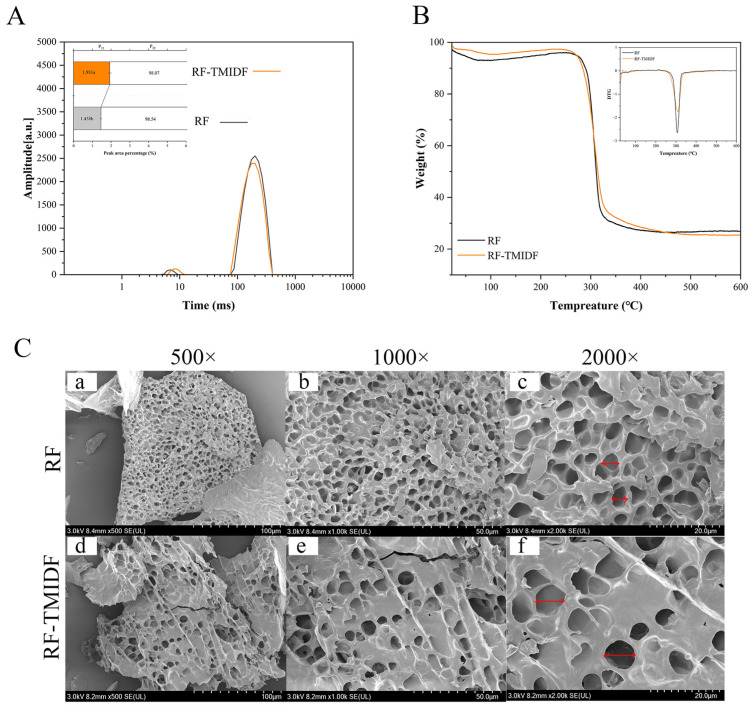
Physicochemical characterization of RF-TMIDF composites. (**A**) Low-field NMR relaxation time spectra of rice flour gels: T_21_, 8.0–13.0 ms (bound water); T_2_, 223.0–291.0 ms (free water); P21/P22, relative peak area proportions of T_21_ and T_22_; (**B**) thermogravimetric profiles (TGA/DTG) of gelatinized samples; (**C**) scanning elcectron micrographs of (a–c) RF and (d–f) RF-TMIDF at magnifications of 500×, 1000×, and 2000×, respectively. Arrows indicate characteristic pore structures.

LF-NMR, a non-invasive technique enabling rapid analysis, was applied to assess the effect of TMIDF on water mobility and distribution within the starch-based system. The transverse relaxation time (T_2_), a parameter highly sensitive to molecular mobility, reflects variations in water binding states and exhibits an inverse correlation with binding strength [28]. As depicted in Figure 2A, the relaxation spectra of all samples displayed two distinct water populations: bound water (T_21_, 8.0–13.0 ms) and free water (T_22_, 63.0–405.0 ms), corresponding to tightly bound water within the gel matrix and more mobile water in less constrained environments, respectively. Notably, upon the incorporation of TMIDF, substantial changes were observed in both the relaxation times and corresponding peak proportions. Specifically, the T_21_ peak shifted rightward (prolonged relaxation time), indicating a stronger binding water, while the bound water proportion (P_21_) increased, and the free water fraction (P_22_) decreased. TMIDF promotion indicates a redistribution of water from free to bound states.

Bound water typically exhibits shorter T_2_ due to strong hydrogen bonding with starch components. The concurrent extension of T_2_ and elevation of P_21_ suggest a fundamental change in water dynamics. This phenomenon suggests that water molecules are not simply experiencing weaker interactions. Instead, they undergo enhanced mobility within a spatially restricted microenvironment. This change is likely the result of TMIDF’s competitive adsorption of water, where TMIDF immobilizes water via physical or chemical interactions [28]. As a result, free water availability for starch granule swelling is reduced, and TMIDF introduces heterogeneous hydration sites with varying degrees of water restriction. These microstructural changes in water distribution provide a mechanistic explanation for the observed macroscopic behavior in the RVA analysis (Table 1). The restricted free water pool limits the extent of amylose recrystallization (dependent on aqueous media), directly correlating with the observed decrease in FV and SB. Concurrently, the densification of the transient gel network during the initial heating phase, driven by competitive water sequestration, accounts for the observed increase in PV. These findings support a mechanistic link between microscopic water dynamics and macroscopic functional properties, reinforcing the role of TMIDF in modulating starch hydration behavior and gelation performance.

### 3.4. Thermal Analysis

The thermal transition parameters of RF with and without TMIDF are summarized in Table 2. The incorporation of 5% TMIDF induced a marked elevation in both the onset temperature (T_o_, from 57.98 ± 0.09 °C to 59.80 ± 0.49 °C) and the peak temperature (T_p_, from 64.82 ± 0.17 °C to 65.57 ± 0.13 °C; *p* < 0.05). This rightward shift in gelatinization temperatures suggests enhanced thermal stability of the starch molecular network, likely due to the structural reinforcement provided by TMIDF. These findings are consistent with the dietary fiber-enhanced starch ordering theory proposed by Wen et al. [17], which posits that DF can promote tighter packing of starch chains through non-covalent interactions. Mechanistically, TMIDF is presumed to competitively adsorb free water molecules, thus reducing the effective hydration of amorphous starch regions. This dehydration effect limits the dissociation of hydrogen bonds during heating, consequently delaying the onset of gelatinization. A similar mechanism has been reported for corn bran fibers in modulating starch–water interactions and thermal behavior [23].

The gelatinization enthalpy change (ΔH), reflecting the energy required for double helix dissociation and crystalline structure disruption, offers insights into the degree of molecular ordering and retrogradation potential. RF-TMIDF exhibited a marked decrease in ΔH values (Table 2), suggesting enhanced thermal stability of crystalline domains, potentially resulting from structural reorganization induced by TMIDF treatment. This phenomenon aligns with decreased mobility of bound water observed in LF-NMR analysis (Figure 2). However, the decreased melting enthalpy (ΔH: 7.62 to 6.97 J/g) in RF-TMIDF might indicate partial destruction of unstable microcrystals during processing. This apparent contradiction likely reflects a trade-off between crystal quality and quantity. The LF-NMR data further support this hypothesis: increased tightly bound water proportion suggests that TMIDF treatment promotes the formation of compact ordered regions. Thus, the reduction in ΔH might not solely indicate thermal weakness but could also reflect a refinement of microstructural features within the starch matrix. Collectively, these results support the hypothesis that TMIDF modifies both the hydration and crystalline behavior of starch, enhancing thermal resistance while introducing subtle reorganizations in starch microstructure.

### 3.5. TGA Analysis

The thermal decomposition profiles of RF with and without TMIDF are summarized in Figure 2B, revealing four distinct degradation stages. Stage I (25–150 °C) corresponds to the evaporation of moisture, primarily driven by the volatilization of free and bound water. In this stage, the RF-TMIDF system exhibited a lower initial mass loss rate than native RF, suggesting a stronger water-retention capability. This observation aligns well with LF-NMR findings (Figure 2A), which indicated increased water immobilization due to hydrogen bonding between TMIDF and water molecules. Stage II (150–220 °C) exhibited minimal mass loss for both systems, indicating the relative thermal stability of starch and DF components prior to significant thermal decomposition. Stage III (220–400 °C) represents the primary degradation phase, characterized by intense thermal cleavage of glycosidic bonds and polysaccharide depolymerization [29]. Interestingly, RF-TMIDF displayed greater mass loss in this stage than RF, which is likely due to the catalytic decomposition of TMIDF-derived β-glucans and cellulose at elevated temperatures. These fibers may undergo catalytic breakdown at elevated temperatures, contributing additional volatiles and degradation byproducts. Stage IV (400–600 °C) involved the carbonization of thermally stable residues. At this stage, RF-TMIDF showed lower final residual mass (25.3%) than RF (27.1%), suggesting a more complete decomposition process. This could be attributed to the formation of a denser molecular network via hydrogen bonding between TMIDF and starch molecules, promoting facilitated uniform thermal degradation [30].

The derivative thermogravimetric (DTG) curves (Figure 2B inset) further quantified differences in thermal decomposition kinetics. The main decomposition peak of RF-TMIDF shifted rightward to a higher value (309 °C) compared to RF (306 °C), accompanied by a reduction in peak intensity. This dual behavior reflects enhanced thermal stability and slower degradation kinetics in the TMIDF-modified system. Two mechanisms likely contribute to this behavior: (1) enhanced hydrogen bonding between fibers and starch delayed molecular chain scission, requiring higher activation energy for decomposition; (2) the rigid β-glucan backbone in TMIDF restricted thermal motion of starch chains, thereby modulating reaction kinetics. These observations are consistent with studies on celery IDF-enhanced glutinous rice thermal stability [31]. Notably, despite reduced residual char content, the delayed degradation profile observed in RF-TMIDF confirms that TMIDF improves the overall thermal resistance and decomposition stability of the composite system.

### 3.6. SEM Analysis

SEM analysis revealed distinct structural modifications in the RF gel network induced by TMIDF incorporation (Figure 2C). The native RF lyophilized gel exhibited a compact, honeycomb-like network at 500× magnification, characterized by uniform pore distribution, smooth surfaces, and distinct particle boundaries (Figure 2C-a). This morphology is consistent with the continuous rigid framework formed during the gelatinization and subsequent retrogradation of unmodified starch [32]. In contrast, the RF-TMIDF composite displayed a thinner, more porous network with enlarged cavities (Figure 2C-d), attributed to enhanced water sublimation pathways during freeze-drying. This can be attributed to TMIDF’s ability to modulate water distribution and promote the formation of water escape channels during the drying process. At higher magnification (1000×), the fibrous morphology of TMIDF became more apparent. Fibers were observed to interweave with starch granules through physical entanglement and localized adhesion, forming a three-dimensional porous matrix (Figure 2C-e). Further magnification (2000×) revealed rough fiber surfaces with micron-scale grooves (Figure 2C-f), likely associated with their high specific surface area and hydroxyl group distribution, which facilitated hydrogen bonding or van der Waals interactions with starch molecules.

Critically, starch granules in the RF-TMIDF matrix showed reduced swelling compared to pure RF, with visible surface depressions and fissures (Figure 2C-f). This observation supports LF-NMR results (Figure 2A), which indicated that TMIDF competes for water via hydroxyl-mediated adsorption, thereby restricting water availability for starch gelatinization. Additionally, the partial encapsulation of starch granules within the fibrous networks (Figure 2C-e) provides a microstructural rationale for the elevated BD observed in RVA analysis (Table 1). The rigid TMIDF framework likely impeded uniform realignment of starch chains under shear stress, exacerbating structural collapse during thermal-mechanical processing. These observations collectively demonstrate TMIDF’s dual role as both a structural modulator and a water competitor in starch-based systems. By disrupting the continuous gel matrix and altering hydration dynamics, TMIDF significantly impacts the microstructure and functional performance of starch-based systems.

### 3.7. FTIR Analysis

**Figure 3:**
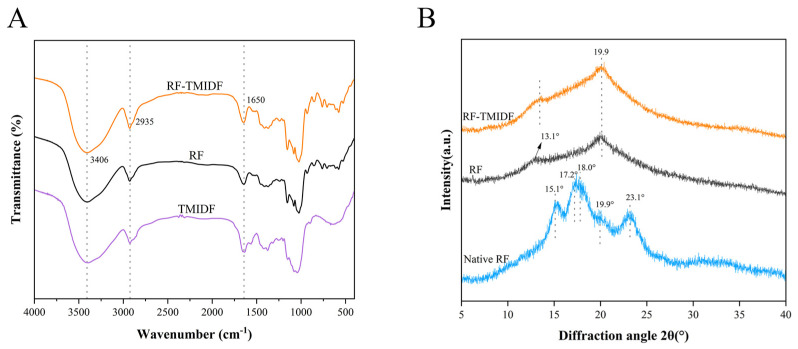
Structural characterization of RF, TMIDF, and their composite (RF-TMIDF). (**A**) FT-IR spectra; (**B**) XRD patterns of native RF and RF-TMIDF.

The FT-IR characteristics of the RF-TMIDF mixture and gelatinized RF are presented in Figure 3A. Compared to pure RF, no additional absorption bands were observed in the TMIDF-incorporated starch gel, indicating that no new chemical bonds formed between TMIDF and RF, with interactions primarily governed by hydrogen bonding. The broad absorption spanning 3000 to 3800 cm^−1^ originates from O–H stretching vibrations. A significant broadening of the absorption band at 3406 cm^−1^ was observed in the gel containing TMIDF. This broadening effect is likely due to the formation of an additional hydrogen bonding network between the free hydroxyl groups of TMIDF polysaccharide chains and the hydroxyl groups of starch molecules, thereby enhancing intermolecular association strength. The band at approximately 1650 cm^−1^ is attributed to the O-H bending vibration of bound water within the sample. Studies indicate that changes in bands within this spectral region are closely related to the water-binding state in the amorphous region of starch [33]. Following TMIDF incorporation, the band at 1650 cm^−1^ exhibited broadening and intensification, suggesting that TMIDF enhances water binding within the system. This finding corroborates Wang et al.’s reported outcomes [34]. Spectral features in the 2800–3000 cm^−1^ region arise from C-H stretching vibrations [13]. Furthermore, the broadening and intensification of the band centered near 2935 cm^−1^ upon TMIDF incorporation provided additional evidence for enhanced intermolecular forces. These results indicate that the introduction of TMIDF, by reinforcing hydrogen bonding interactions, promotes the reorganization of the hydrated structure within the starch amorphous region and strengthens intermolecular interactions between starch chains. In summary, TMIDF alters the microstructure of starch by enhancing hydrogen bonding interactions, thereby contributing to the observed modifications in thermal and rheological properties.

### 3.8. XRD Analysis

The XRD patterns in Figure 1B reveal distinct changes in crystallinity in the RF induced by TMIDF incorporation. Native RF exhibited characteristic peaks at 2θ values of 15.1°, 17.2°, 18.0°, and 23.1°, indicative of a hybrid A-type crystalline structure [35]. Upon gelatinization, the A-type polymorph was disrupted, and new characteristic peaks corresponding to the V-type crystalline structure appeared at 13.1° and 19.9°(2θ). These align with characteristic reflections of amylose-lipid complexes. Notably, TMIDF addition induced significant enhancement of peak intensities at 13.1° and 19.9° compared to control RF (Figure 3B), without generating new diffraction features. This indicates (i) TMIDF does not alter the fundamental crystalline assembly type of starch [11]. (ii) TMIDF promotes denser molecular packing within amylose-lipid assemblies. This enhancement likely originates from the high crystallinity of IDF. TMIDF promotes denser packing of amylose-lipid assemblies through a physical restriction effect, which simultaneously limits the mobility of amylose chains and suppresses disordered realignment. Similar phenomena have been reported for insoluble fibers with high crystallinity [36]. Furthermore, the XRD-derived trends in crystallinity correspond well with the altered gelatinization behavior detected in DSC measurements (Table 2). This dual-scale modification yields a starch-fiber composite with improved thermal stability and structural integrity.

### 3.9. α-Amylase Inhibition Rate Analysis

**Figure 4:**
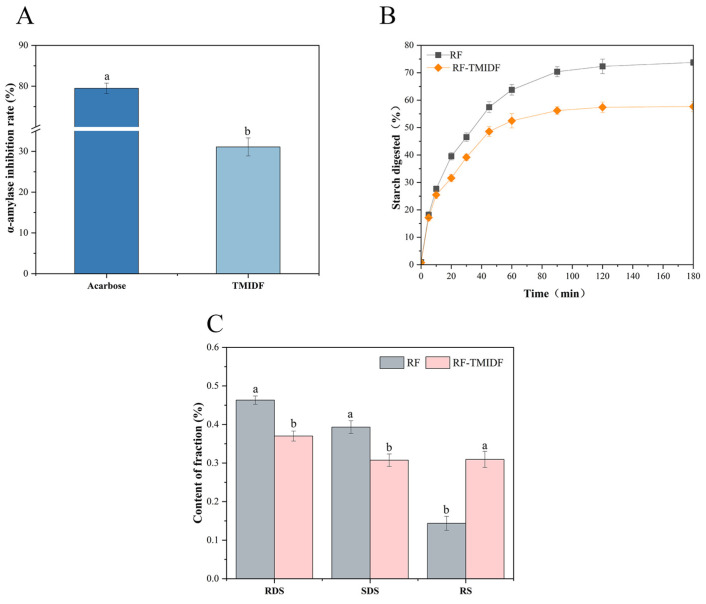
Effects of TMIDF on RF digestion. (**A**) Inhibition rates of α-amylase activity by TMIDF and acarbose (positive control); (**B**) digestive kinetics of RF and RF-TMIDF over time; (**C**) contents of RDS, SDS, and RS in RF and RF-TMIDF. W the same group, a and b indicate significant differences (*p* < 0.05).

As a key digestive enzyme catalyzing the breakdown of starch and glycogen, α-amylase plays a central role in determining the rate of carbohydrate hydrolysis and postprandial glucose response. This study demonstrated that TMIDF exhibited 31.08% α-amylase inhibition (Figure 4A), indicating its potential to attenuate starch digestion. Although this inhibition level was lower than that of the pharmaceutical inhibitor acarbose (79.47%), it falls within the range commonly observed for plant-derived IDFs [37]. The inhibition mechanism likely involves TMIDF’s porous network and high specific surface area, which physically adsorb α-amylase molecules [38] and sterically hinder enzyme-substrate binding [39]. Specifically, when α-amylase and starch are embedded within the TMIDF matrix, enzymatic accessibility to starch is reduced due to restricted diffusion and competitive adsorption [40]. Similar effects have been reported for burdock IDF, which also leverages fibrous architectures to suppress glycemic responses [37]. These findings highlight the potential of fungal-derived dietary fibers, such as TMIDF, as functional ingredients for developing dietary strategies targeting glycemic control.

### 3.10. In Vitro Digestion Analysis

The incorporation of 5% TMIDF significantly modulated starch digestion kinetics in RF, as evidenced by the delayed hydrolysis profile (Figure 4B) and elevated RS content (Figure 4C). Compared to native RF, the final starch digestibility of RF-TMIDF decreased from 74.74% to 57.65%, while RS content increased markedly from 14.36% to 30.94%. During the initial rapid hydrolysis phase (0–30 min), corresponding to enzymatic degradation of amorphous starch regions [41], TMIDF suppressed digestion through synergistic mechanisms: physical barriers formed by its porous fibrous network (SEM) restricted enzyme accessibility despite increased surface area, while enhanced V-type crystallinity (XRD) impeded α-amylase penetration. These structural modifications align with reports highlighting dietary fiber-starch interactions as key regulators of glycemic control [25].

The pronounced RS enrichment in RF-TMIDF stems from three interconnected mechanisms: (i) encapsulation of partially gelatinized starch granules within the TMIDF matrix, creating enzyme-inaccessible zones; (ii) enzymatic resistance of V-type amylose-lipid complexes [42]; and (iii) direct inhibition of α-amylase activity [43]. This multi-layered defense strategy (comprising structural reinforcement, enzyme inhibition, and diffusion control) offers a more comprehensive modulation of starch digestibility than the single-mechanism approach typical of conventional plant IDFs. As such, TMIDF represents a promising dietary component for designing functional food systems aimed at sustained glucose release and improved glycemic regulation.

## 4. Conclusions

This study systematically elucidates the multifunctional role of TMIDF in modulating the pasting behavior, structural properties, and digestibility of RF. Incorporation of 5% TMIDF significantly enhanced the peak viscosity and shear resistance of RF during gelatinization, attributed to its competitive water adsorption and physical entanglement with starch chains, which restricted granule swelling while promoting transient network densification. Concurrently, TMIDF suppressed short-term retrogradation (reduced setback viscosity) by hindering amylose realignment through hydrogen bonding and steric effects. Multi-scale characterization revealed that TMIDF induced hierarchical structural modifications: (i) SEM and XRD analyses demonstrated the formation of a porous fibrous network and enhanced V-type crystallinity, respectively, which collectively impeded enzyme accessibility; (ii) LF-NMR and DSC data highlighted water redistribution and improved thermal stability driven by TMIDF-starch interactions; (iii) FT-IR confirmed the dominance of non-covalent interactions in stabilizing the composite system. Crucially, TMIDF reduced in vitro starch digestibility by 22.9% and elevated RS content by 115%, achieved through synergistic mechanisms—physical encapsulation of starch granules, enzymatic resistance of V-type amylose-lipid complexes, and direct α-amylase inhibition. These findings transcend the conventional single-barrier mechanism of plant-derived IDFs, proposing a novel “structural optimization-enzyme suppression-diffusion control” paradigm for starch digestion regulation. The dual functionality of TMIDF (enhancing processing performance while improving nutritional attributes) positions it as a promising candidate for designing low GI functional foods. This work not only advances the understanding of fungal IDF-starch interactions but also provides a sustainable strategy for valorizing underutilized medicinal-edible fungi in the food industry. However, this study also has some limitations, including the evaluation of only a single TMIDF concentration (5%) and reliance on an in vitro digestion model, which may not fully replicate physiological conditions. Future studies should extend these findings by (1) exploring dose-dependent effects of TMIDF, (2) validating its glycemic impact through in vivo studies, and (3) assessing its performance in complex food matrices to better optimize its application in metabolic health management.

## Figures and Tables

**Table 1:** Pasting properties of RF and RF-TMIDF.

Sample	PT (°C)	PV (mPa·s)	TV (mPa·s)	FV (mPa·s)	BD (mPa·s)	SB (mPa·s)
RF	67.53 ± 0.25 ^a^	2573.21 ± 31.29 ^b^	1015.12 ± 28.45 ^a^	1998.27 ± 52.19 ^a^	1558.09 ± 58.50 ^b^	986.48 ± 21.70 ^a^
RF-TMIDF	67.16 ± 0.45 ^a^	2814.52 ± 18.03 ^a^	1017.23 ± 21.70 ^a^	1886.18 ± 31.74 ^b^	1807.28 ± 9.22 ^a^	848.94 ± 47.27 ^b^

PT, pasting temperature; PV, peak viscosity; TV, trough viscosity; FV, final viscosity; BD, breakdown viscosity; SB, setback viscosity. Values represent mean ± SD (n = 3). Different superscript lowercase letters (a, b) within a column denote significant differences (*p* < 0.05; Student’s *t*-test).

**Table 2:** Thermal properties and short-range order analysis of RF and RF-TMIDF composite system.

Sample	T_o_ (°C)	T_p_ (°C)	T_c_ (°C)	ΔH (J/g)
RF	57.98 ± 0.09 ^b^	64.82 ± 0.17 ^b^	70.82 ± 0.10 ^b^	7.62 ± 0.27 ^a^
RF-TMIDF	59.80 ± 0.49 ^a^	65.57 ± 0.13 ^a^	71.26 ± 0.30 ^a^	6.97 ± 0.25 ^b^

Thermal properties were characterized by onset (T_o_), peak (T_p_), and conclusion (T_c_) temperatures, alongside gelatinization enthalpy (ΔH). Values (mean ± SD, n = 3) with distinct superscritts within columns differ significantly (*p* < 0.05).

## Data Availability

The original contributions presented in the study are included in the article, further inquiries can be directed to the corresponding author.
